# Species Delimitation of the *Eisenia nordenskioldi* Complex (Oligochaeta, Lumbricidae) Using Transcriptomic Data

**DOI:** 10.3389/fgene.2020.598196

**Published:** 2020-12-07

**Authors:** Sergei V. Shekhovtsov, Aleksandra A. Shipova, Tatiana V. Poluboyarova, Gennady V. Vasiliev, Elena V. Golovanova, Anna P. Geraskina, Nina A. Bulakhova, Tímea Szederjesi, Sergei E. Peltek

**Affiliations:** ^1^Department of Molecular Biotechnology, Institute of Cytology and Genetics of the Siberian Branch of the Russian Academy of Sciences, Novosibirsk, Russia; ^2^Kurchatov Genomic Center, Institute of Cytology and Genetics of the Siberian Branch of the Russian Academy of Sciences, Novosibirsk, Russia; ^3^Laboratory of Biocenology, Institute of Biological Problems of the North of the Far Eastern Branch of the Russian Academy of Sciences, Magadan, Russia; ^4^Laboratory of Systematics and Ecology of Invertebrates, Omsk State Pedagogical University, Omsk, Russia; ^5^Center for Forest Ecology and Productivity of the Russian Academy of Sciences, Moscow, Russia; ^6^Laboratory of Biodiversity and Ecology, Tomsk State University, Tomsk, Russia; ^7^Department of Zoology, Hungarian Natural History Museum, Budapest, Hungary

**Keywords:** phylogenomics, phylogeny, transcriptomes, Lumbricidae, *Eisenia nordenskioldi*, earthworms

## Abstract

*Eisenia nordenskioldi* (Eisen, 1879) is the only autochthonous Siberian earthworm with a large distribution that ranges from tundra to steppe and broadleaved forests. This species has a very high morphological, ecological, karyological, and genetic diversity, so it was proposed that *E. nordenskioldi* should be split into several species. However, the phylogeny of the complex was unclear due to the low resolution of the methods used and the high diversity that should have been taken into account. We investigated this question by (1) studying the diversity of the COI gene of *E. nordenskioldi* throughout its range and (2) sequencing transcriptomes of different genetic lineages to infer its phylogeny. We found that *E. nordenskioldi* is monophyletic and is split into two clades. The first one includes the pigmented genetic lineages widespread in the northern and western parts of the distribution, and the second one originating from the southern and southeastern part of the species' range and representing both pigmented and non-pigmented forms. We propose to split the *E. nordenskioldi* complex into two species, *E. nordenskioldi* and *Eisenia* sp. 1 (aff. *E. nordenskioldi*), corresponding to these two clades. The currently recognized non-pigmented subspecies *E. n. pallida* will be abolished as a polyphyletic and thus a non-natural taxon, while *Eisenia* sp. 1 will be expanded to include several lineages earlier recognized as *E. n. nordenskioldi* and *E. n. pallida*.

## Introduction

*Eisenia nordenskioldi* (Eisen, 1879) is the only autochthonous Siberian earthworm with a large distribution, which includes the whole Siberia from the steppe zone in the south to certain Arctic islands, as well as the East European plain, parts of Kazakhstan, China, Korea, and Mongolia (Perel, 1979; Vsevolodova-Perel, 1997). This species was found to contain a very high morphological, ecological, and karyotypic diversity (Viktorov, 1997; Vsevolodova-Perel and Bulatova, 2008; Blakemore, 2013). *E. nordenskioldi* is traditionally divided into two subspecies, *E. n. nordenskioldi* and *E. n. pallida*, the latter differing solely by the absence of pigmentation (Malevich, 1956; Vsevolodova-Perel, 1997). Blakemore (2013) recently added two more subspecies, *E. n. mongol* and *E. n. onon*, based on single locations in Mongolia.

DNA sequence data (Blakemore, 2013; Shekhovtsov et al., 2013, 2015, 2016a,b, 2017b, 2018a,b; Hong and Csuzdi, 2016) demonstrated that genetic diversity within *E. nordenskioldi* is extremely high. Different genetic lineages of *E. nordenskioldi* can differ by as much as 22% of nucleotide substitutions in the mitochondrial COI gene (Shekhovtsov et al., 2013). Such level of divergence is typically characteristic for distinct congeneric species, or even different genera within a family (Hebert et al., 2003). This diversity is not only found in geographically remote populations: individuals identified as *E. nordenskioldi*, but highly diverged on the DNA level are often found in the same soil sample (Shekhovtsov et al., 2013, 2018b).

This newly discovered genetic diversity was characterized either as new subspecies (Blakemore, 2013) or as “genetic lineages” (Shekhovtsov et al., 2013, 2016a). Genetic lineage is an established term among earthworm scientists (King et al., 2008; Martinsson and Erséus, 2017; Marchán et al., 2018) that refers to intraspecific clades that are highly genetically diverged from other clades within the species. At least 15 distinct lineages are currently recognized within *E. nordenskioldi*, and deep genetic divergence between some of them were verified by the phylogenomic approach (Shekhovtsov et al., 2019). On the whole, the gathered evidence implies that this taxon encompasses several distinct species. However, phylogenetic relationships among the recognized subspecies and genetic lineages of *E. nordenskioldi* remain unclear.

Studies based on single mitochondrial and nuclear genes (Shekhovtsov et al., 2013, 2016a) suggested that both of the subspecies of *E. nordenskioldi* may be polyphyletic. However, this evidence could not be considered decisive due to the few DNA loci involved in the analysis. An attempt to resolve the phylogeny of the complex using mitochondrial genomes (Shekhovtsov et al., 2020) proved to be insufficient to clarify some questions. When using different datasets (i.e., protein sequences, protein-coding genes, rRNAs, total genomes, etc.), *E. nordenskioldi* was recovered either as mono- or as paraphyletic. Relationships among different lineages were also inconsistent. Extensive multigene data on nuclear sequences is obviously needed to resolve these issues.

The aim of this study was to determine the phylogeny of the *E. nordenskioldi* complex and to clarify its systematics. To do this, we sequenced transcriptomes of 12 *E. nordenskioldi* specimens belonging to different subspecies and genetic lineages. We assembled protein and DNA sequence datasets to construct a multigene phylogeny of the complex. In addition, we sequenced the COI gene of multiple geographically remote populations and combined those with the data obtained earlier in order to estimate the diversity within the complex throughout its distribution. We also investigated the validity of the closely related species *E. atlavinyteae* and the unpigmented subspecies *E. n. pallida*. Based on this data, we propose the ways this complex could be split into natural taxa.

## Materials and Methods

### Earthworm Sampling and DNA Sequencing

**Figure 1:**
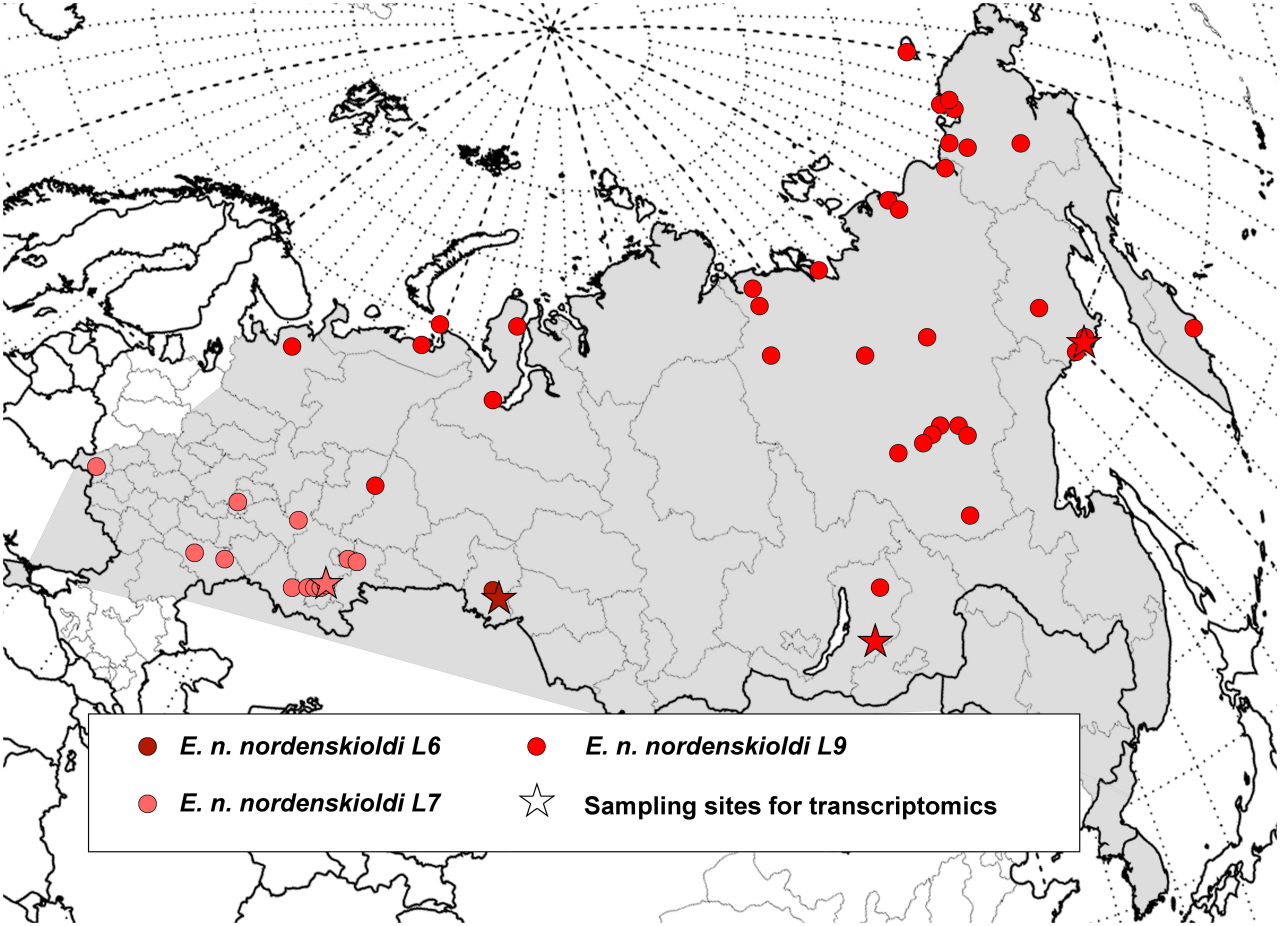
Geographic distribution of the genetic lineages of *E. nordenskioldi*. Distribution of the *E. nordenskioldi* complex is shown in gray. Color and symbol code refers to Figures 2–4.

**Figure 2:**
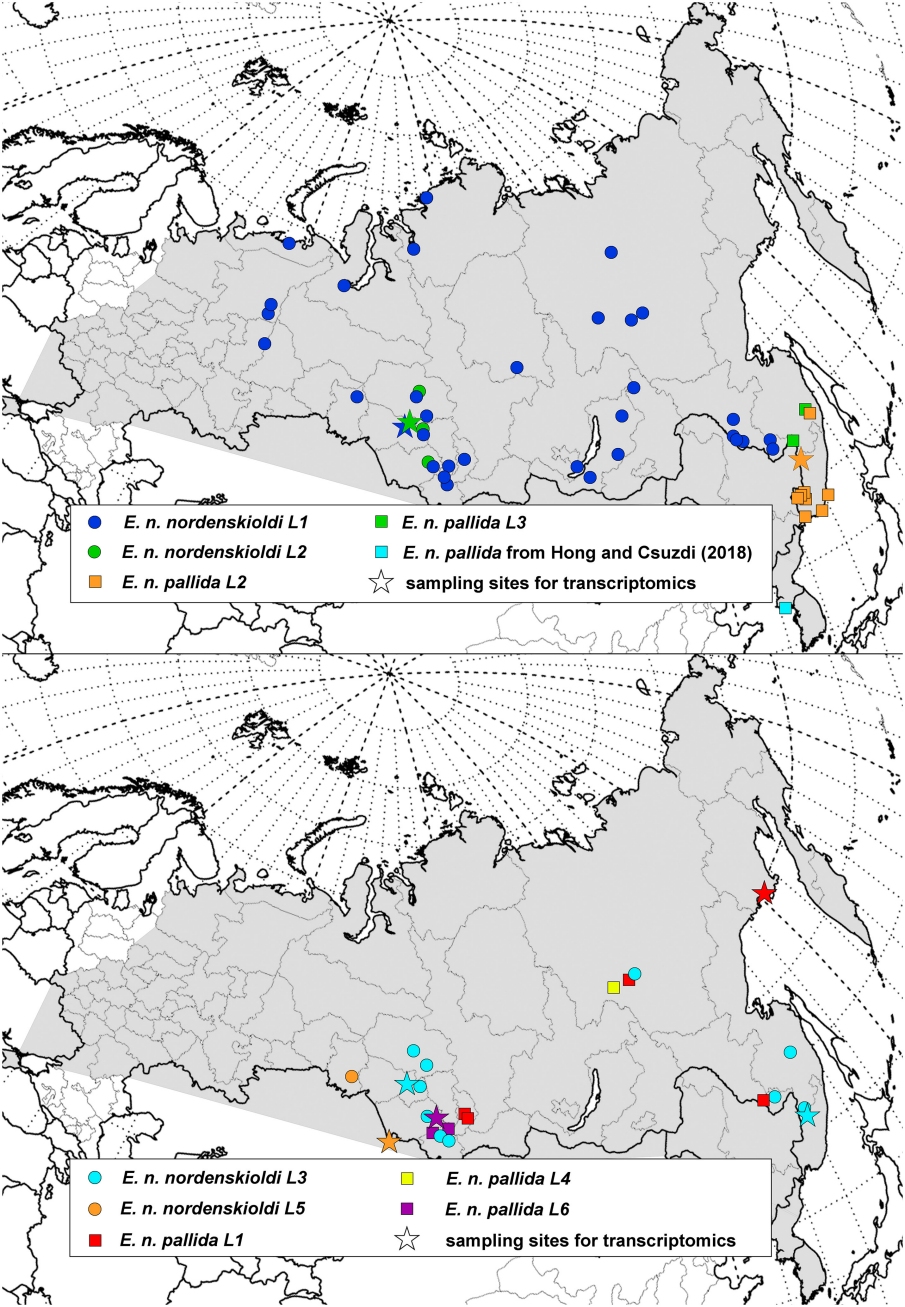
Geographic distribution of the genetic lineages of *Eisenia* sp. 1. Distribution of the *E. nordenskioldi* complex is shown in gray. Color and symbol code refers to Figures 1, 3, 4.

For phylogeographic analysis, we took the ethanol-fixed specimens we collected in 2011–2020, as well as those provided by colleagues (Figures 1, 2). Earthworms were identified according to the key of Vsevolodova-Perel (1997). A section of the posterior body end was excised for DNA isolation using the BioSilica columns (BioSilica, Russia) according to the manufacturer's instructions. The fragment of the mitochondrial COI gene was amplified using the universal LCO and HCO primers (Folmer et al., 1994). The obtained amplicons were purified with the exonuclease I—shrimp alkaline phosphatase mix (New England Biolabs, USA) and sequenced using the BigDye 3.1 (Applied Biosystems, USA). Only the new and unique haplotypes (a total of 62) were submitted to the GenBank under accession nos. MT863028–MT863089.

For transcriptomic analysis, live earthworm individuals were collected in 2016–2019 (see Table 1). RNA was extracted by the Trizol method as described in Shekhovtsov et al. (2019). For large worms, RNA was extracted from a section of the body containing the clitellum; for small individuals, the whole body was used. A small part of the tail was fixed in ethanol and used for DNA extraction and COI fragment sequencing as described above. The COI barcode was used to identify the genetic affinity of the specimens against our dataset of *Eisenia* sequences. Individuals belonging to the desired genetic lineage were taken for transcriptome analysis.

**Table 1:** Samples taken for transcriptomic analysis.

**Sample**	**Sampling location**	**Reads**	**Bases**	**Ntr**	**N50**	**nr**	**Portho**	**HaMStR**
*E. n. n. L1*	Russia, Buryatia Republic, Ukyr	6,829,313	893,561,003	66,734	796	19,584	16,092	1,060
*E. n. n. L2*	Russia, Novosibirsk oblast, Novosibirsk	5,046,196	662,670,274	65,367	611	17,792	14,535	969
*E. n. n. L3*	–//–	1,002,477	273,164,798	41,724	830	17,469	13,195	941
*E. n. n. L3*	Russia, Khabarovsk krai, Lesopil'noye	22,060,622	2,984,499,927	144,485	1,093	36,889	21,345	1,146
*E. n. n. L5*	Kazakhstan, East Kazakhstan oblast, Semipalatinsk	8,065,857	1,069,397,358	92,936	729	27,110	19,577	1,144
*E. n. n. L6*	Russia, Omsk oblast, Omsk	4,858,412	642,125,976	63,101	655	16,371	13,436	941
*E. n. n. L7*	Russia, Bashkortostan Republic, Baymak district, Faizullino	901,691	233,570,944	26,230	787	12,617	10,087	851
*E. n. n. L9*	Russia, Magadan oblast, Magadan	907,172	267,977,445	35,751	693	13,952	10,946	876
*E. n. n. L9*	Russia, Buryatia Republic, Ukyr	4,811,412	645,880,417	53,376	699	12,641	10,416	869
*E. n. pallida L1*	Russia, Magadan oblast, Magadan	549,677	217,418,513	20,730	576	7,013	5,537	565
*E. n. pallida L2*	Russia, Khabarovsk krai, Lesopil'noye	8,398,708	1,115,244,684	84,739	867	24,763	19,265	1,100
*E. n. pallida L6*	Russia, Altai krai, Makarievka	5,696,720	770,053,754	67,896	711	18,664	15,123	1,016
*E. tracta*	Kazakhstan, East Kazakhstan oblast, Gornaya Ul'binka	1,863,378	248,862,467	21,011	574	4,650	3,986	554
*E. magnifica*	–//–	3,542,634	471,425,980	35,149	604	7,817	6,469	662
*E. balatonica*	Kazakhstan, East Kazakhstan oblast, Semipalatinsk	3,326,331	442,401,654	37,999	651	9,186	7,821	761
*E. spelaea*	Hungary, Köszeg, Pogányok	2,676,755	348,105,082	31,509	501	6,627	5,340	638
*E. andrei*	Russia, Novosibirsk oblast, Novosibirsk	1,094,366	195,074,949	41,886	811	19,351	12,929	974

*E. n. n., Eisenia n. nordenskioldi; Ntr, number of assembled transcripts; nr, number of non-redundant proteins; POrtho, number of the detected ProteinOrtho orthogroups; HaMStR, number of the detected HaMStR orthogroups*.

### Transcriptome Sequencing and Assembly

RNA was purified using the PureLink RNA Micro Kit (Invitrogen, USA). RNASeq library preparations were carried out with 1 μg of total RNA using the TruSeq Stranded mRNA LT Sample Prep Kit (Illumina, USA) according to the manufacturer's instructions with small modifications (4 min RNA fragmentation time and 12 PCR cycles). The quantity and quality of the libraries were assessed using the Agilent Bioanalyzer 2100 System and DNA High sensitivity kit (Agilent Technologies, USA). After normalization, barcoded libraries were pooled and sequenced on the NextSeq550 sequencer using the NextSeq 550 Mid Output v2 Kit 150 cycles (Illumina, USA).

Transcriptome *de novo* assembly was performed using the Trinity v.2.4.0 (Grabherr et al., 2011) with the default parameters. Protein coding sequences were predicted by the TransDecoder v.5.0.0 (http://transdecoder.github.io). Redundant proteins were removed by the CD-HIT v.4.6 (Fu et al., 2012) at the 99% similarity threshold. Mitochondrial sequences were filtered out using a custom script (https://github.com/sashulkaSh/worms/blob/main/script.sh).

### Dataset Construction and Phylogenetic Analysis

Datasets of orthologous groups were constructed using two programs that employ different algorithms. ProteinOrtho v.5.16b (Lechner et al., 2011) was used to search for related proteins present in all studied transcriptomes with the 1e−30 e-value threshold. HaMStR v.13.2.6 (Ebersberger et al., 2009) detected only the proteins annotated in two reference genomes, *Helobdella robusta* and *Capitella* sp. Functional annotation of the predicted orthologous groups was performed by mapping the abovementioned Pfam and Swiss-Prot hits to the Gene Ontology (GO) categories using the built-in Swiss-Prot information and pfam2go data (http://www.geneontology.org/external2go/pfam2go). In both cases, only the proteins present in all 17 studied transcriptomes were retained in the final datasets.

The detected protein sequences were aligned by the MAFFT v.7.394 (Katoh and Standley, 2013). Gaps and ambiguously aligned portions of the alignment were discarded using the TrimAl v.1.4.rev22 (Capella-Gutiérrez et al., 2009) with the “strictplus” and “nogaps” options. Protein alignments shorter than 20 amino acids were removed. To avoid the impact of outliers, we discarded alignments with dissimilarities between any pair of sequences outside the upper 95th percentile. The rest were filtered using the PhyloTreePruner v.1.0 (Kocot et al., 2013) on the corresponding ML trees generated with the FastTreeMP v.2.1.7 (Price et al., 2010).

The obtained protein datasets were taken into phylogenetic analysis. We also created nucleotide datasets by extracting transcript data for each of the proteins from the datasets described above. For each gene, the transcripts were aligned by the MAFFT with the “-adjustdirection” option. When several isoforms were detected, the longest one was used. Alignments shorter than 30 bp were discarded.

For phylogenetic tree construction, the Maximum Likelihood (ML) analysis was performed in RAxML (Stamatakis, 2014). The most appropriate substitution models were selected based on the Bayesian information criterion as implemented in the MEGA X (Kumar et al., 2018): JTT for the ProteinOrtho amino acid dataset, JTT+I+G for the HaMStR amino acid dataset, and GTR+I+G for all nucleotide datasets. For each dataset, 1,000 bootstrap replicates were performed. Bayesian inference was done by the MrBayes v. 3.4 (Ronquist et al., 2012) with the same substitution models. For each dataset, 10,000,000 generations were run in two independent analyses from different random starting trees; 25% of generations were discarded as burn-in.

For automated species delimitation, the ABGD algorithm (Puillandre et al., 2012) was used. This analysis was run online at https://bioinfo.mnhn.fr/abi/public/abgd/. Another method used was the multi-rate Poisson Tree Process (Kapli et al., 2017; http://mptp.h-its.org/#/tree) with the ML phylogenetic tree as the input. The ancestral area reconstruction analysis was performed using the S-DIVA (Statistical dispersal-vicariance analysis) implemented in the RASP v. 4 (Yu et al., 2015, 2020).

## Results

### Transcriptome Sequencing

In this study, we performed low-depth Illumina singe-end transcriptomes for 12 specimens of *E. nordenskioldi* and outgroup species, and combined this data with five Ion Torrent transcriptomes from our earlier study (Shekhovtsov et al., 2019). The final dataset contained transcriptomes for 12 *E. nordenskioldi* lineages, as well as five outgroup species of the genus *Eisenia*. Sequence statistics on the studied transcriptomes is given in Table 1. The number of reads for different libraries ranged from 0.5 to 22 million reads. Sequences statistics are summarized in Table 1. *De novo* assembly by Trinity produced 21–144 thousand transcripts with N50 ranging from 500 to 1,000.

Two different algorithms were used to construct datasets for phylogenetic analysis. The algorithm implemented in ProteinOrtho found 287 common genes for the 17 specimens (212 after filtering). The final amino acid alignment contained 29,097 amino acids; nucleotide alignment contained 107,394 nucleotide positions. HaMStR detected 227 common genes, but only 91 remained after filtration. The resulting alignments contained 7,309 amino acids and 35,917 nucleotides, respectively.

The average pairwise distances between *E. nordenskioldi* lineages are provided in Supplementary Table 1. For the COI dataset, these distances ranged from 15 to 19%, negligibly lower than between them and *E. andrei*. For the ProteinOrtho nucleotide dataset, distances between the lineages were 2.5–5.5%, which was significantly lower than between them and *E. andrei* (8.2–8.6%).

### Multigene Phylogeny of the *E. nordenskioldi* Complex

**Figure 3:**
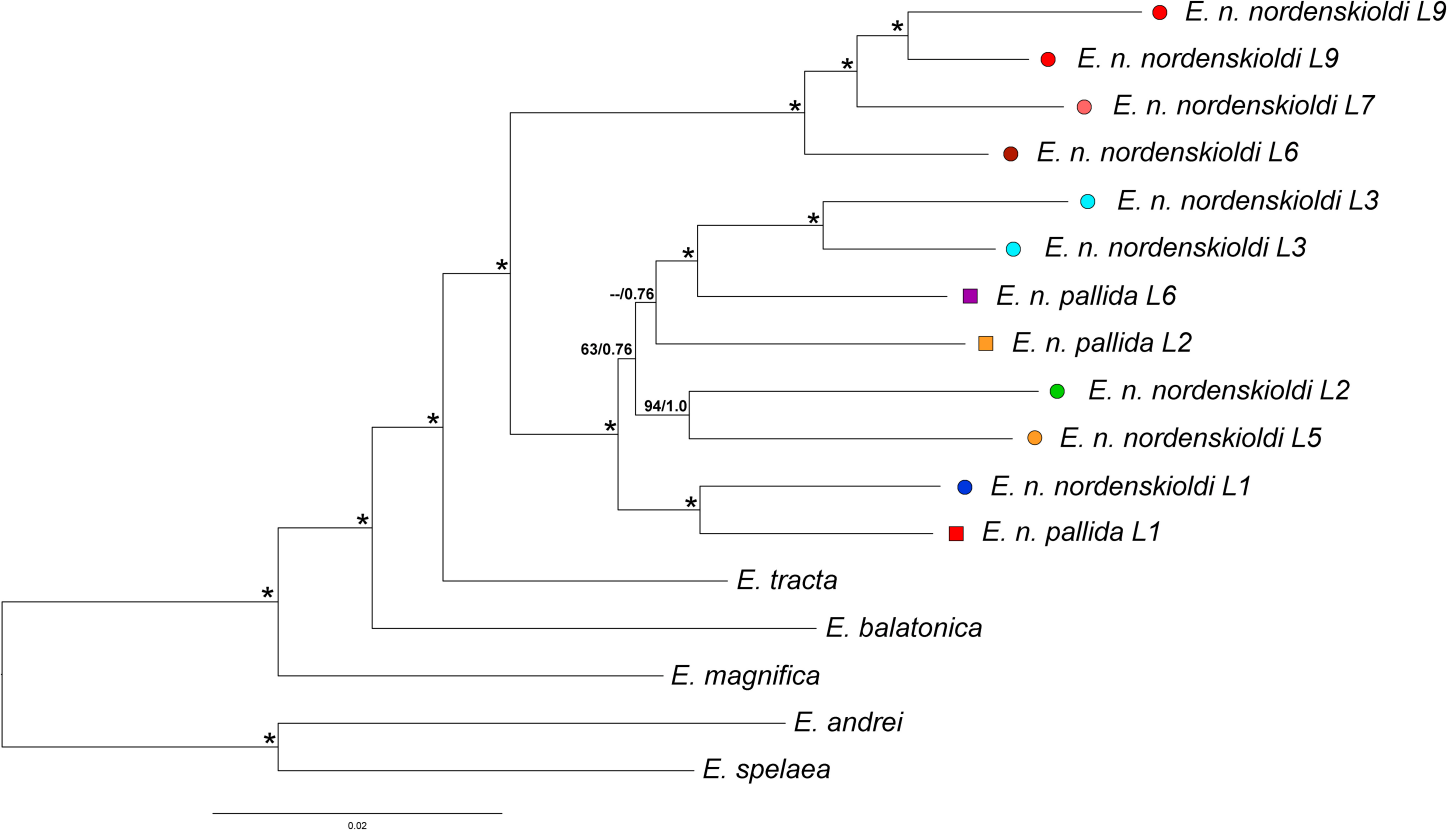
Phylogenetic tree for the nucleotide ProteinOrtho dataset constructed using the ML algorithm. Numbers near branches indicate ML bootstrap support/Bayesian posterior probabilities; asterisks stand for 100/1.0. Lineage symbols refer to Figures 1, 2, 4.

Phylogenetic trees constructed using both algorithms had similar topologies (Figure 3). The studied species of the genus *Eisenia* were divided into two groups, one containing European species [*E. spelaea* (Rosa, 1901) and *E. andrei* (Bouché, 1972)], and the other, specimens from Siberia [*E. nordenskioldi, E. magnifica* (Svetlov, 1957), *E. tracta* (Perel, 1985), and *E. balatonica* (Pop, 1943)]. *E. tracta* was found to be the sister species of *E. nordenskioldi*. *E. nordenskioldi* invariably split into two groups. One of those comprised *E. n. nordenskioldi* lineages 6, 7, and 9, and the other, all *E. n. pallida* lineages and the rest of *E. n. nordenskioldi*.

Within the second group, the recovered phylogenetic relationships were not completely resolved. *E. n. nordenskioldi* lineage 1 and *E. n. pallida* lineage 1 clustered together. Another groups that were invariably recovered were *E. n. nordenskioldi* lineages 2 and 5, and *E. n. nordenskioldi* lineage 3 and *E. n. pallida* lineage 6. However, relationships among those clades was unstable; *E. n. nordenskioldi* lineage 1/*E. n. pallida* lineage 1 was recovered as a sister group to the rest of the lineages, but with low statistical support (Figure 3).

### *E. nordenskioldi* Haplotype Distribution and Automated Species Delimitation

We assembled a dataset containing 927 COI barcode sequences of *E. nordenskioldi* specimens. Of those, 252 were newly sequenced, and the rest was taken from previous studies (Shekhovtsov et al., 2013, 2015, 2016a,b, 2018a, 2020). Haplotypes of *E. n. pallida* lineage 5 were excluded from the analysis because it was demonstrated that this is probably a species of the *Aporrectodea* genus similar in morphology to *E. n. pallida* (Shekhovtsov et al., 2016a) and it did not group together with the rest of *E. nordenskioldi* on phylogenetic trees (not shown).

**Figure 4:**
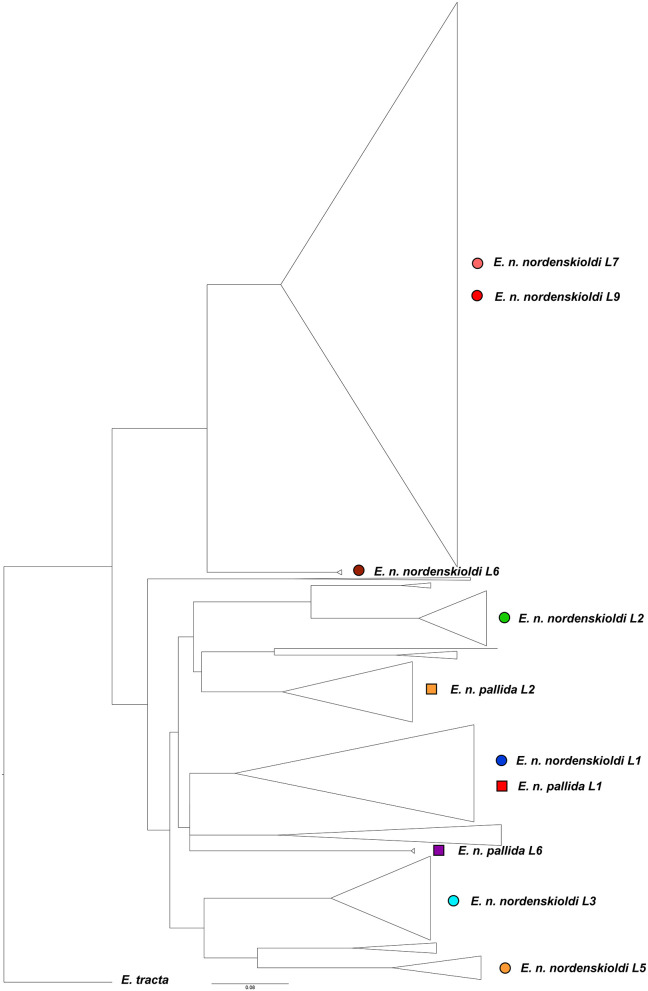
Phylogenetic tree for the COI dataset constructed using the ML algorithm. Lineage symbols refer to Figures 1–3.

The final sample contained 373 full-length unique haplotypes. No indels were detected. Of the total 658 nucleotide positions, 284 were variable and 263 were parsimony-informative. Phylogenetic tree for this dataset (Figure 4) confirmed the division of *E. nordenskioldi* into two groups. One can also see that the majority of genetic lineages reported in earlier studies were detected, as well as several new ones. *E. n. nordenskioldi* lineages 7 and 9 are usually recovered as separate clades, but here, lineage 7 was nested within lineage 9. *E. n. nordenskioldi* lineage 1 and *E. n. pallida* lineage 1 also merged into a single clade.

The obtained data was used to construct distribution maps. Short COI sequences, as well as those with poorly read fragments were assigned to one of the detected *E. nordenskioldi* lineages. This was also applied to the data from Blakemore (2013) and Hong and Csuzdi (2016). The sequences of *E. n. mongol* and *E. n. onon* (Blakemore, 2013) were nested within *E. n. nordenskioldi* lineage 1, close to certain haplotypes from the Buryatia Republic, while those of *E. n. pallida* from Korea (Hong and Csuzdi, 2016) formed a separate branch within *Eisenia* sp. 1 (not shown).

We attempted to divide *E. nordenskioldi* into putative species by automated species delimitation using various algorithms. The multi-rate Poisson tree processes analysis (Kapli et al., 2017) detected 77 putative species. The ABGD algorithm (Puillandre et al., 2012) suggested 29 to 46 species with the default parameters (relative gap width = 1.5X) and 8–12 species with the 4X relative gap width. Boundaries of these species were consistent with those drawn manually for *E. nordenskioldi* (Figure 4; Shekhovtsov et al., 2013, 2018a,b). Pairwise differences between the studied lineages for the COI gene are high, in most cases ranging from 15 to 19% (Supplementary Table 1).

The ancestral area reconstruction analysis (Supplementary Figure 1) suggested that the areas of the *E. nordenskioldi* complex and of the two main clades found within were located in the south of West Siberia, most probably in the Ob and Irtysh basins.

## Discussion

### Phylogeny of the *E. nordenskioldi* Complex

Phylogenomic analysis performed in this study allowed us to provide definitive answers to several issues. Although complete mitochondrial genomes suggested *E. nordenskioldi* to be paraphyletic with respect to *E. tracta* (Shekhovtsov et al., 2020), nuclear datasets unequivocally recover *E. nordenskioldi* as monophyletic. We also confirmed that neither *E. n. nordenskioldi* nor *E. n. pallida* are monophyletic.

*E. nordenskioldi* was found to be split into two large clades. One of those includes haplotypes belonging to the earlier recognized genetic lineages 6, 7, and 9 of *E. n. nordenskioldi* (Figure 3), and the other, to the rest of the specimens recognized as *E. n. nordenskioldi*, as well as those belonging to *E. n. pallida*. Comparison of the phylogenomic tree (Figure 3) and the COI tree (Figure 4) demonstrates that the phylogenomic datasets cover the majority of *E. n. nordenskioldi* diversity, and that most phylogenetic groupings coincide among those trees, although the COI tree provides far less resolution.

### Current Delimitation of *E. nordenskioldi*

It was long known that *E. nordenskioldi* has extreme morphological, karyological, and ecological diversity (Malevich, 1956; Perel, 1979; Graphodatsky et al., 1982; Malinina and Perel, 1984; Viktorov, 1997; Shekhovtsov et al., 2017a). There were several attempts to split *E. nordenskioldi* into smaller taxa. The non-pigmented form was denoted as *E. nordenskioldi* forma *pallida* by Malevich (1956) and later on given the status of subspecies by Vsevolodova-Perel (1997). The obvious problem here is that these two forms are often sympatric (Vsevolodova-Perel and Leirikh, 2014), while subspecies have to be allopatric according to the conventional definition (Mayr, 1982). Vsevolodova-Perel probably felt that differences in pigmentation only without any other diagnostic characters did not justify the status of a separate species, so the recognition of *E. n. pallida* as a sympatric subspecies became established.

In this study, we found that *E. n. pallida* is unequivocally polyphyletic. Genetic lineages of *E. n. pallida* from different regions belong to different lineages and represent independent cases of pigmentation loss, probably as a consequence of the shift to the endogenic lifestyle.

Perel and Graphodatsky (1984) described two species, *E. atlavinyteae* and *E. sibirica*, earlier regarded as morphological forms slightly deviating from the typical diagnosis. *E. atlavinyteae* differed from *E. nordenskioldi* by the clitellum starting on the 26th segment, and by the *tubercula pubertatis* always present on the 28th segment. However, the authors noted that this arrangement was observed only in a portion of the type population, while in the rest, the position of these characters was close to those characteristic for *E. nordenskioldi*. *E. sibirica* had the clitellum spanning the segments 27–32, but again with significant intrapopulation variation overlapping the diagnosis of *E. nordenskioldi* (Perel and Graphodatsky, 1984).

These two newly described species were thus hard to distinguish from *E. nordenskioldi* in practice. In this study, we collected a set of individuals throughout the species' range identified as *E. atlavinyteae* from different locations. However, they did not form a separate clade and turned out to belong to lineages 1 and 3 of *E. nordenskioldi*. *E. atlavinyteae*, thus, does not represent a separate species and probably represents a morphological form that in some populations makes up the majority of individuals. Individuals identified as *E. sibirica* from a population from Western Siberia were found to belong to *E. nordenskioldi* lineage 2, along with those having the typical *E. nordenskioldi* diagnosis.

We can thus conclude that none of the major currently recognized taxa within *E. nordenskioldi* or related to it represent monophyletic clades: *E. n. nordenskioldi* is paraphyletic, *E. n. pallida* is polyphyletic, and *E. atlavinyteae* and *E. sibirica* probably represent morphological forms, not distinct species. The question on the latter two, however, cannot be considered as concluded without genetic investigation of type specimens.

### Delimitation of the *E. nordenskioldi* Complex

As shown in the previous section, the current delimitation of the species complex is not consistent with its phylogeny. It is generally accepted that the newly described taxa should be monophyletic, however, criteria for species delimitation in earthworms are otherwise vague. One way to propose these putative species is to compare the extent of genetic variation among the detected clades and within them. Several algorithms of automated species delimitation exist. The two programs used in this study identifies from eight to as many as 77 species. One can see that the species' sampling is currently sufficiently dense (Figures 1, 2), so the observed deep split is not the result of sparse sampling of highly diverged haplotypes.

Although we could isolate these as new species or subspecies, splitting the species into so many new cryptic taxa would be highly impractical. We propose to divide *E. nordenskioldi* into two species. *E. nordenskioldi* was described by Eisen (1879) based on a set of populations from the Vaigach island and along the lower reaches of the Yenisei. Vaigach Island can be thus considered the type location. It is known to be inhabited by lineage 9, so we suggest that one of the detected clades including *E. n. nordenskioldi* lineages 6, 7, and 9 to be denoted as *E. nordenskioldi*.

The second clade on Figure 3 contained multiple clades corresponding to both pigmented and non-pigmented lineages. Phylogenetic relationships among them were only partially resolved, suggesting rapid diversification. Many of these clades have overlapping distributions and are found in sympatry (Figure 2) while being genetically distinct, and so might be viewed as distinct species. However, we suggest to isolate this whole clade as a putative new species, *Eisenia* sp. 1 (aff. *E. nordenskioldi*). Preliminary results of the morphological analysis suggest that *E. nordenskioldi* and *Eisenia* sp. 1 have some detectable differences (Golovanova et al., unpublished), which would allow a formal description of this species.

## Conclusions

Phylogenomic data suggests that the *E. nordenskioldi* complex is split into two clades, one containing pigmented genetic lineages from the northern and western part of the distribution, and the second, both pigmented and non-pigmented ones mostly from the south and the southeast of the species' range. The existence of the two subspecies, *E. n. nordenskioldi* and *E. n. pallida*, as well as of the related species *E. atlavinyteae* and *E. sibirica* was not supported by the genetic analysis. We propose to split the *E. nordenskioldi* complex into two species corresponding to the abovementioned clades: the northern and western lineages as *E. nordenskioldi*, and the southern and southeastern ones, as *Eisenia* sp. 1 (aff. *E. nordenskioldi*).

## Data Availability Statement

The datasets presented in this study can be found in online repositories. The names of the repository/repositories and accession number(s) can be found at: https://www.ncbi.nlm.nih.gov/, PRJNA658219; https://www.ncbi.nlm.nih.gov/genbank/, MT863028–MT863089.

## Author Contributions

SS designed the study. SS, GV, NB, and TS collected and identified the specimens. SS and TP extracted RNA. GV performed RNA sequencing. AS extracted sequence datasets. SS built phylogenetic trees, analyzed the data, and wrote the paper with input from AS, GV, and SP. All authors read and approved the final manuscript.

## Conflict of Interest

The authors declare that the research was conducted in the absence of any commercial or financial relationships that could be construed as a potential conflict of interest.
