# Supplementary figures and images for: Species Delimitation of the Eisenia nordenskioldi Complex (Oligochaeta, Lumbricidae) Using Transcriptomic Data
Source: Front Genet. 2020 Dec 7;11:598196. doi: 10.3389/fgene.2020.598196 (PMC7750196; doi:10.3389/fgene.2020.598196)

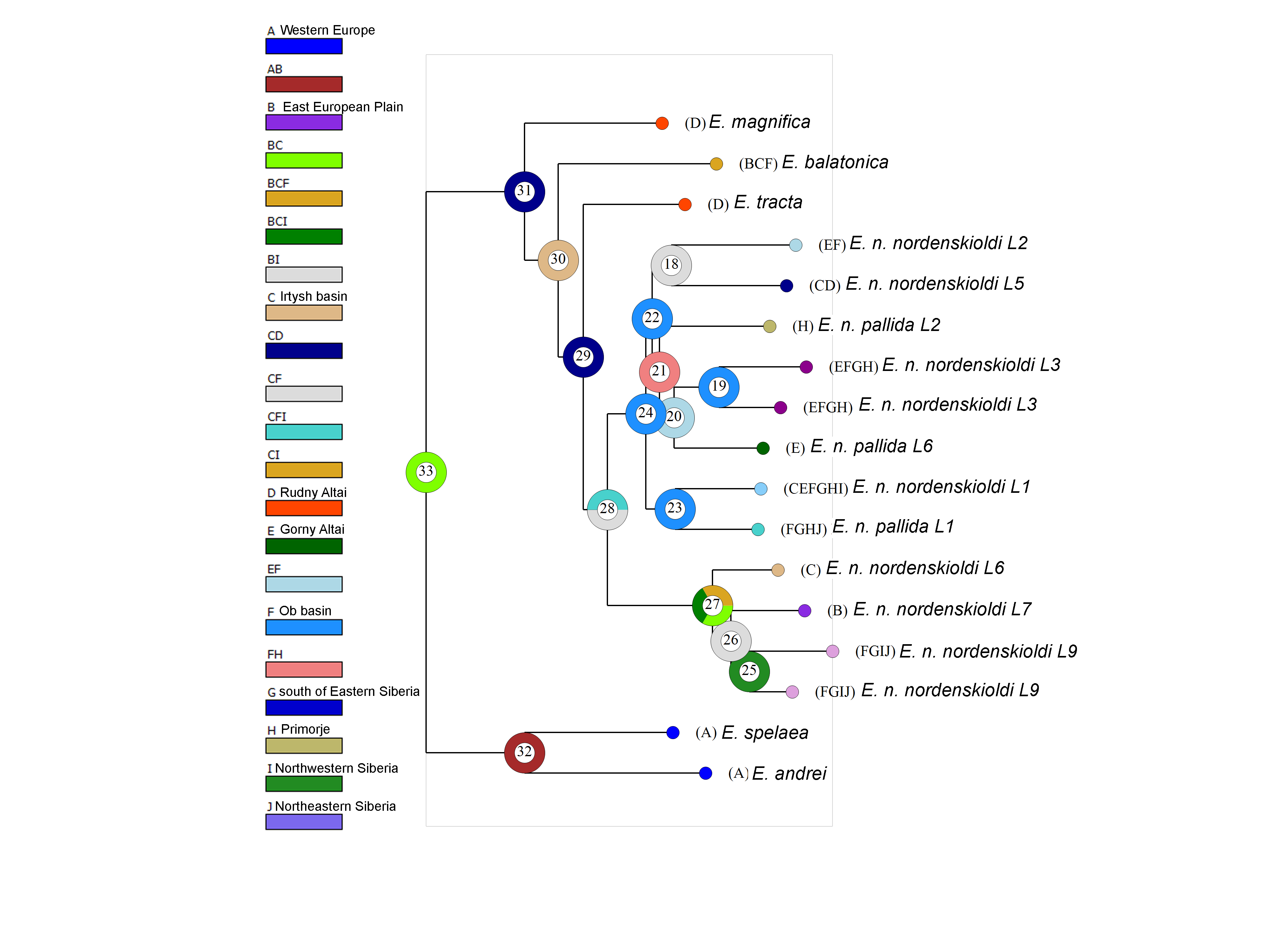

Supplement: Supplementary file 1 [file Image_1.tif]
