# Supplementary material for: Species Delimitation of the Eisenia nordenskioldi Complex (Oligochaeta, Lumbricidae) Using Transcriptomic Data
Source: Front Genet. 2020 Dec 7;11:598196. doi: 10.3389/fgene.2020.598196 (PMC7750196; doi:10.3389/fgene.2020.598196)
Supplement: Supplementary file 2 [file Table_1.docx]

SUPPLEMENTARY TABLE 1. Pairwise p-distances (in %) among the studied genetic lineages of *E. nordenskioldi*. N, *E. n. nordenskioldi* lineages; P, *E. n. pallida* lineages. Below diagonal, for the COI sample; above diagonal, ProteinOrtho nucleotide dataset.

|  | N6 | N7 | N9 | N1 | N2 | N3 | N5 | P1 | P2 | P6 | *E.andrei* |
| --- | --- | --- | --- | --- | --- | --- | --- | --- | --- | --- | --- |
| N6 | - | 2,59 | 2,73 | 4,96 | 5,09 | 5,11 | 5,04 | 4,73 | 4,95 | 4,76 | 8,35 |
| N7 | 14,21 | - | 2,64 | 5,25 | 5,35 | 5,29 | 5,16 | 4,93 | 5,17 | 4,97 | 8,59 |
| N9 | 13,59 | 9,68 | - | 5,29 | 5,50 | 5,43 | 5,24 | 5,07 | 5,24 | 5,02 | 8,74 |
| N1 | 17,33 | 18,20 | 17,88 | - | 4,08 | 3,98 | 3,91 | 2,89 | 3,61 | 3,58 | 8,42 |
| N2 | 18,80 | 18,17 | 18,57 | 18,09 | - | 3,94 | 3,98 | 3,72 | 4,00 | 3,74 | 8,48 |
| N3 | 17,75 | 17,79 | 18,83 | 17,34 | 18,05 | - | 4,04 | 3,77 | 3,95 | 3,38 | 8,54 |
| N5 | 18,38 | 18,55 | 18,42 | 15,13 | 18,53 | 18,09 | - | 3,71 | 3,84 | 3,72 | 8,55 |
| P1 | 16,01 | 17,23 | 17,58 | 15,04 | 20,30 | 17,93 | 17,26 | - | 3,64 | 3,41 | 8,29 |
| P2 | 16,58 | 17,98 | 17,94 | 16,23 | 17,80 | 16,49 | 16,94 | 11,90 | - | 3,33 | 8,38 |
| P6 | 17,48 | 17,73 | 17,04 | 14,13 | 18,21 | 15,68 | 18,04 | 15,96 | 16,59 | - | 8,25 |
| *E.andrei* | 17,38 | 19,35 | 19,32 | 19,69 | 20,54 | 20,17 | 20,40 | 19,60 | 19,14 | 19,00 | - |
